# Use of a panel of four microRNAs in CSF as a predicted biomarker for postoperative neoangiogenesis in moyamoya disease

**DOI:** 10.1111/cns.13646

**Published:** 2021-05-04

**Authors:** Gang Wang, Yunyu Wen, Siyuan Chen, Guozhong Zhang, Mingzhou Li, Shichao Zhang, Songtao Qi, Wenfeng Feng

**Affiliations:** ^1^ Department of Neurosurgery Nanfang Hospital Southern Medical University Guangzhou China

**Keywords:** angiogenesis, biomarker, cerebrospinal fluid (CSF), indirect bypass surgery, moyamoya disease (MMD), prediction model

## Abstract

**Introduction and Aims:**

At present, the treatment for moyamoya disease (MMD) primarily consists of combined direct and indirect bypass surgery. Nevertheless, more than half of indirect bypass surgeries fail to develop good collaterals from the dura and temporal muscle. This study aimed to investigate whether microRNAs (miRNAs) in cerebrospinal fluid (CSF) could serve as biomarkers for the prediction of postoperative collateral formation.

**Methods:**

Moyamoya disease patients with indirect bypass surgery were divided into angiogenesis and non‐angiogenesis groups, CSF was obtained, and miRNA sequencing was performed using the CSF. Candidate miRNAs were filtered and subsequently verified through qRT‐PCR. The diagnostic utility of these differential miRNAs was investigated by using receiver operating characteristic (ROC) curve analysis. Finally, the potential biological processes and signaling pathways associated with candidate miRNAs were analyzed using R software.

**Results:**

The expression levels of four miRNAs (miR‐92a‐3p, miR‐486‐3p, miR‐25‐3p, and miR‐155‐5p) were significantly increased in the angiogenesis group. By combining these four miRNAs (area under the curve [AUC] =0.970), we established an accurate predictive model of collateral circulation after indirect bypass surgery in MMD patients. GO and KEGG analyses demonstrated a high correlation with biological processes and signaling pathways related to angiogenesis.

**Conclusion:**

The 4‐miRNA signature is a good model to predict angiogenesis after indirect bypass surgery and help the surgeon to select a appreciate bypass strategy.

## Graphical abstract

According to the establishment of collaterals, Moyamoya disease (MMD) patients who suffered direct bypass and indirect bypass were divided into two group, obtained their cerebrospinal fluid (CSF), and screened the small RNA sequencing, bioinformatics analysis for all differential miRNAs, and filtering the candidate miRNAs. For validated of candidate miRNAs, qRT‐PCR was further used to analyzed the miRNAs which differential expression in two group. Then, ROC curve analysis was performed to evaluate the diagnostic value of the four identified miRNAs for the establishment of collaterals. To further understand the potential biological functions of the four miRNAs, we identified the functions of target genes using GO and KEGG pathway enrichment analyses.

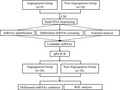

## 1 INTRODUCTION

Moyamoya disease (MMD) is a progressive cerebrovascular disorder manifesting stenosis or occlusion of a terminal portion of the internal carotid artery (ICA) or a proximal portion of the anterior cerebral arteries and the middle cerebral arteries (ACAs, MCAs), as well as the development of abnormal vascular networks at the skull base. In adult patients, intracranial hemorrhage, as well as cerebral ischemia, cognitive dysfunction, epilepsy, involuntary movement, and headache, are frequently observed.^1^, ^2^ To treat MMD, bypass surgery is recommended, including direct superficial temporal to middle cerebral artery bypass (STA‐MCA) and indirect bypass, such as encephalomyosynangiosis (EMS), encephalomyomyosynangiosis (EDMS), and encephalo‐duro‐arteriosynangiosis (EDAS), as well as the combined strategy.^3^ Unlike direct bypass surgery, which can provide immediate augmentation of blood flow, indirect surgery takes more time to introduce angiogenesis from the muscle and dura. Many studies have indicated that CB is more effective than indirect surgery in preventing recurrent stroke in adults.^4^, ^5^ However, unfortunately, nearly half of adult moyamoya patients exhibited poor potential for transdural angiogenesis after combined bypass.^6^ For this group of MMD patients, adequate anastomosis did not develop between the ischemic brain tissue and the capillary‐rich grafts, such as the temporal muscle or dura mater, after the combined surgery. In this condition, indirect bypass surgery was ineffective for preventing stroke attack but increased the risk of such complications as temporal muscle‐related mass effects, seizures, and big bite ischemic phenomena.^7^, ^8^, ^9^ These complications could be avoided by abandoning combined bypass if we could predict the patient's potential for collateral formation preoperatively. Younger age at operation was associated with good postoperative collateral formation.^10^, ^11^ Preoperative transdural collaterals could be utilized as radiographic biomarkers to predict better long‐term angiographic results following surgery. Abundant ICA moyamoya vessels indicate good angiogenesis. However, the molecular mechanism regulating angiogenesis after indirect bypass surgery has not been elucidated.^12^ A reliable biomarker with a high degree of sensitivity and specificity may represent a complementary and cost‐effective means of helping surgeons determine which MMD patient is more suitable for indirect bypass procedures.

MicroRNAs (miRNAs) are short noncoding RNAs that can bind to the 3’‐untranslated regions (3’‐UTRs) of target mRNAs and subsequently play important roles in nearly all biological processes; miRNAs are associated with many diseases, such as cardiovascular disease, stroke, aneurysm, and moyamoya disease.^13^, ^14^, ^15^, ^16^, ^17^ Studies have shown that miRNAs regulate angiogenesis directly by influencing the activity of endothelial cells or indirectly by modulating the expression of proteins that promote or inhibit vessel growth.^18^, ^19^ MiRNAs have a dual action in angiogenesis.^17^ Fang et al. demonstrated that miR‐29b suppresses tumor angiogenesis, invasion, and metastasis by regulating matrix metalloproteinase 2 expression.^20^ Chen et al. demonstrated that increasing miR‐126‐5p expression in the temporal muscle can promote endothelial cell proliferation and angiogenesis in chronically ischemic brains of two‐vessel occlusion plus EMS rats through the PI3K/Akt pathway.^21^

In this study, we hypothesized that miRNAs in CSF are potential biomarkers in MMD patients to predict angiogenesis after indirect bypass surgery. CSF miRNAs were systematically screened by using miRNA arrays and validated by miRNA real‐time PCR. Bioinformatic analyses identified several important angiogenesis‐related pathways and CSF miRNAs that may be involved in the postoperative collateral formation process.

## 2 MATERIALS AND METHODS

### 2.1 Patients and samples collection

From October 2017 to December 2019, 60 patients (31 males and 29 females) diagnosed with moyamoya disease by digital subtraction angiography (DSA) in Nanfang Hospital were admitted to this study. Briefly, the CSF of 20 patients, including 10 angiogenesis groups and 10 non‐angiogenesis groups, was used for small RNA sequencing. Because one sample failed in library construction, the actual execution was 19 samples in small RNA sequencing. The CSF of another 40 patients, including 20 angiogenesis groups and 20 non‐angiogenesis groups, was used for RT‐PCR. The diagnostic criteria for MMD were based on the guidelines published in 2012 by the Research Committee on the Spontaneous Occlusion of the Circle of Willis of the Ministry of Health and Welfare, Japan. All patients underwent STA‐MCA bypass surgery and encephalo‐duro‐myo‐synangiosis (EDMS). Specific surgical techniques were described as previously described.^22^ A 20‐ml syringe was used to obtain 10 ml CSF after opening the dura during the first surgery. The CSF was placed in a 15‐ml RNase/DNase‐free centrifuge tube. The whole CSF was centrifuged at 1,500 g for 10 min at room temperature, and the supernatant was transferred to a 15‐ml RNase/DNase‐free centrifuge tube. The specimens were then stored at −80°C. DSA was used to assess the establishment of collateral circulation at 6 months after the operation. Postsurgical angiogenesis was evaluated by using the Matsushima standard. If the collaterals distributed more than one‐third of the middle cerebral artery, the collaterals were assigned to the angiogenesis group; if slight or none, the collaterals were assigned to the non‐angiogenesis group. The study was approved by the Review Committee of the Nanfang Hospital Ethics Committee of Southern Medical University (NFEC‐201906‐K11), and informed consent was obtained from the patients.

### 2.2 RNA extraction and quality control

Total RNA from CSF was extracted using the miRNeasy Serum/Plasma Kit (QIAGEN, NO. 217184) according to the manufacturer's protocol. Quantitation of total RNA was performed using an Agilent 2100 Bioanalyzer (Agilent Technology, USA). Quality‐controlled RNA was employed for subsequent RNA sequencing and quantitative PCR (qPCR).

### 2.3 RNA‐sequencing analysis and bioinformatic analysis

One nanogram of total RNA from each sample was used for small RNA library construction using TruSeq Small RNA Sample Prep Kits (Cat. No. RS‐200–0012, Illumina, USA) following the manufacturer's recommendations. In brief, total RNA was ligated to adapters at each end. Next, the adapter‐ligated RNA was reverse‐transcribed to cDNA, and PCR amplification was performed. PCR products ranging from 140 to 160 bp were isolated and purified as small RNA libraries. Library quality was assessed with the Agilent Bioanalyzer 2100 system using DNA High Sensitivity Chips. The libraries were finally sequenced using the Illumina HiSeq X Ten platform. Small RNA sequencing and analysis were conducted by OE Biotech Co., Ltd. (Shanghai, China).

The basic reads were converted into sequence data by base calling. Low‐quality reads were filtered for subsequent analysis. The known miRNAs were identified by alignment against the miRBase v22 database (http://www.mirbase.org/), and the known miRNA expression patterns in different samples were analyzed. The age was considered as a covariance by using the R package RUVSeq in the following analysis. Differential expressed miRNAs were calculated and filtered with the threshold of *p* value <0.05 and |Fold change| >2. The targets of miRNAs were predicted by using four prediction websites: TargetScan, miRDB, miRTarbase, and TargetMiner. Summarize all downstream genes predicted by the above website. Then, difference integration analysis (Venn analysis) was performed.

### 2.4 Candidate miRNAs screening

The miRNAs whose transcripts per million (TPM) >10 in at least one group from differentially expressed known miRNAs were selected. Subsequently, miRNAs with target genes that are significantly correlated with angiogenesis were selected as candidate biomarkers and subsequently subjected to quantitative validation.

### 2.5 MiRNA quantitative PCR

Quantification was performed with a two‐step reaction process: reverse transcription (RT) and PCR. Each RT reaction consisted of 4.5 μl RNA, 5 μl of 2X TS miRNA Reaction Mix and 0.5 μl of TransScrip miRNA RT Enzyme Mix in a total volume of 10 μl. Reactions were performed in a GeneAmp^®^ PCR System 9700 (Applied Biosystems, USA) for 60 min at 37°C followed by heat inactivation at RT for 5 s at 85°C. Real‐time PCR was performed using a LightCycler^®^ 480 II Real‐time PCR Instrument (Roche, Switzerland) with a 10‐μl PCR mixture that included 1 μl of cDNA, 5 μl of 2X PerfectStart^TM^ Green qPCR SuperMix, 0.2 μl of universal primer, 0.2 μl of miRNA‐specific primer, and 3.6 μl of nuclease‐free water. Reactions were incubated in a 384‐well optical plate (Roche, Switzerland) at 94°C for 30 s followed by 45 cycles of 94°C for 5 s and 60°C for 30 s. Each sample was run in triplicate for analysis. The expression levels of miRNAs were normalized to 5S rRNA and calculated using the 2^−ΔΔCt^ method (Livak and Schmittgen, 2001). All primers are listed in Table S1.

### 2.6 ROC curve analysis

Receiver operating characteristic (ROC) curves were constructed to assess the predictive value of the candidate miRNAs by calculating the AUCs in MMD patients. The sensitivity and specificity of the miRNA prognostic signature in predicting clinical outcome were evaluated by calculating the AUC value of the ROC curve using Prism 7.0 software.

### 2.7 Statistical analysis

The bubble phases were determined using R software (version 3.5.1), and the ROC curve and summary data were generated using GraphPad Prism version 7 and 8 (San Diego, California USA). Venny 2.1.0 (https://bioinfogp.cnb.csic.es/tools/venny/) was used to determine the distribution of the differentially expressed miRNAs and mRNAs. The results are presented as means ± SEM, and statistical analysis was performed between the angiogenesis group and the non‐angiogenesis group using Student's *t* test in SPSS 24.0. *p* value <0.05 was considered to be significant.

## 3 RESULTS

### 3.1 Characteristics of the study population

**FIGURE 1:**
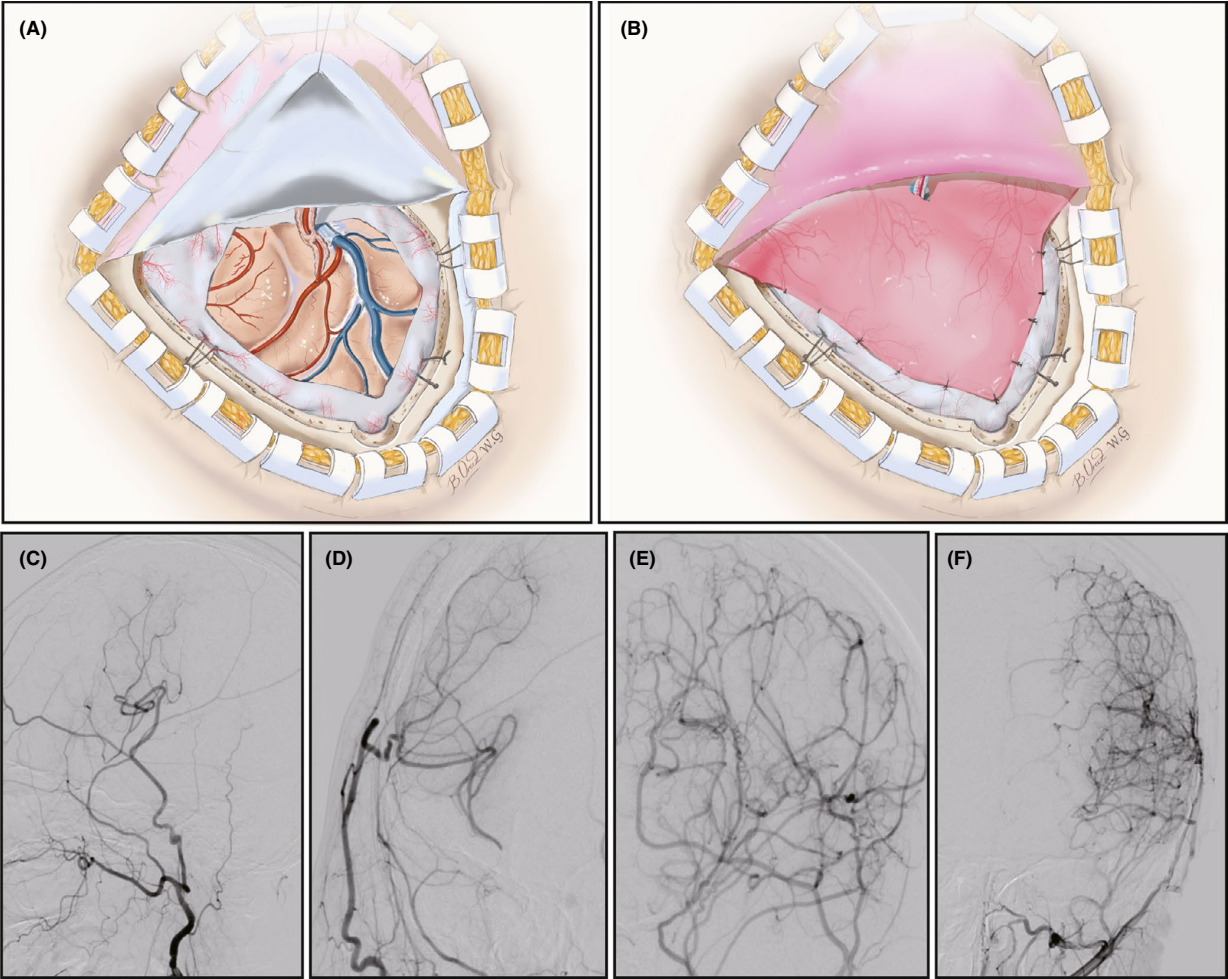
The schematic diagram of bypass surgery and evaluation of postsurgical angiogenesis by using the Matsushima standard. (A–B) The scope of EMS surgery. (C–D) Non‐angiogenesis group: no obvious collateral formation. (C) Side view, (D) Front view. (E–F) Angiogenesis group: abundant collateral formation. (E) Side view, (F) Front view

Figure 1A,B outline the surgical technique of EDMS. MMD patients were divided into two groups according to whether there was collateral circulation: the non‐angiogenesis group (Figure 1C,D) and the angiogenesis group (Figure 1E,F). The baseline characteristics of the two groups in the screening and validation sets are listed in Table 1. Liu et al. and Yu et al. have indicated that the leptomeningeal collateral (LMC) status is highly predictable for postoperative angiographic outcomes.^23^, ^24^ According to their evaluation methods, there was no difference in LMC grade between the two groups. There were no significant differences in the distribution of most routine blood biochemical parameters, such as Hb (*p* = 0.777) and Glu (*p* = 0.828), between the two groups in either the screening or validation sets. However, significant differences were observed in the age (*p* < 0.001) levels.

**TABLE 1:** Clinical characteristics of study population (29 angiogenesis group patients and 30 non‐angiogenesis group patients)

Characteristics	Angiogenesis	Non‐angiogenesis	*p* value
Number	29	30	
Gender			
Male	14 (48.3%)	17 (56.7%)	0.52
Female	15 (51.7%)	13 (43.3%)	
Age	35.5 ± 15.7	47.3 ± 8.0	<0.001
Matsushima grade			
I	12 (41.4%)	8 (26.7%)	0.223
II	3 (10.3%)	1 (3.3%)	
III	5 (17.2%)	7 (23.3%)	
IV	3 (10.3%)	1 (3.3%)	
V	0 (0%)	0 (0%)	
VI	6 (20.7%)	13 (43.3%)	
Symptom			
Normal	0 (0%)	6 (20%)	0.123
Dizziness	9 (31.0%)	8 (26.7%)	
Headache	7 (24.1%)	4 (13.3%)	
Muscle weakness	11 (37.9%)	9 (30%)	
Consciousness disorder	2 (6.9%)	3 (10%)	
LMC grade			
Good	22 (75.9%)	23 (76.7%)	0.942
Poor	7 (24.1%)	7 (23.3%)	
Blood examination			
WBC(×10^9^)	8.0 ± 3.3	6.4 ± 1.4	0.022
RBC(×10^9^)	4.6 ± 0.7	4.6 ± 0.7	0.773
Hb(g/L)	131.6 ± 14.9	130.4 ± 18.7	0.777
Glu(mmol/L)	4.9 ± 0.5	4.8 ± 0.6	0.828
HbA1c(mmol/L)	5.6 ± 0.7	6.0 ± 0.9	0.173
K+(mmol/L)	4.1 ± 0.3	3.9 ± 0.3	0.087
Na+(mmol/L)	140.6 ± 2.8	141.0 ± 2.5	0.548
ALT(U/L)	23.5 ± 19.5	22.2 ± 13.8	0.753
AST(U/L)	19.0 ± 8.4	17.4 ± 5.9	0.422
TC(mg/DL)	190.2 ± 121.3	164.3 ± 30.8	0.429
TG(mg/DL)	129.1 ± 55.9	142.5 ± 113.8	0.669
HDL(mg/DL)	41.3 ± 9.0	40.1 ± 9.6	0.711
LDL(mg/DL)	99.2 ± 28.0	102.6 ± 22.0	0.706
APTT(s)	29.0 ± 5.9	27.9 ± 4.9	0.456
PT(s)	10.9 ± 1.4	10.6 ± 1.0	0.348

Abbreviations: ALT, Alanine aminotransferase; APTT, Activated partial thromboplastin time; AST, Aspartate aminotransferase; Ghb, Glycated hemoglobin; Glu, Glucose; Hb, Hemoglobin; HDL, High‐density lipoprotein; LDL, Low‐density lipoprotein; LMC, Leptomeningeal collateral; PT, Prothrombin time; RBC, Red blood cell; TC, Total cholesterol; TG, Triglyceride; WBC, White blood cell.

### 3.2 Principal component analysis (PCA)

**FIGURE 2:**
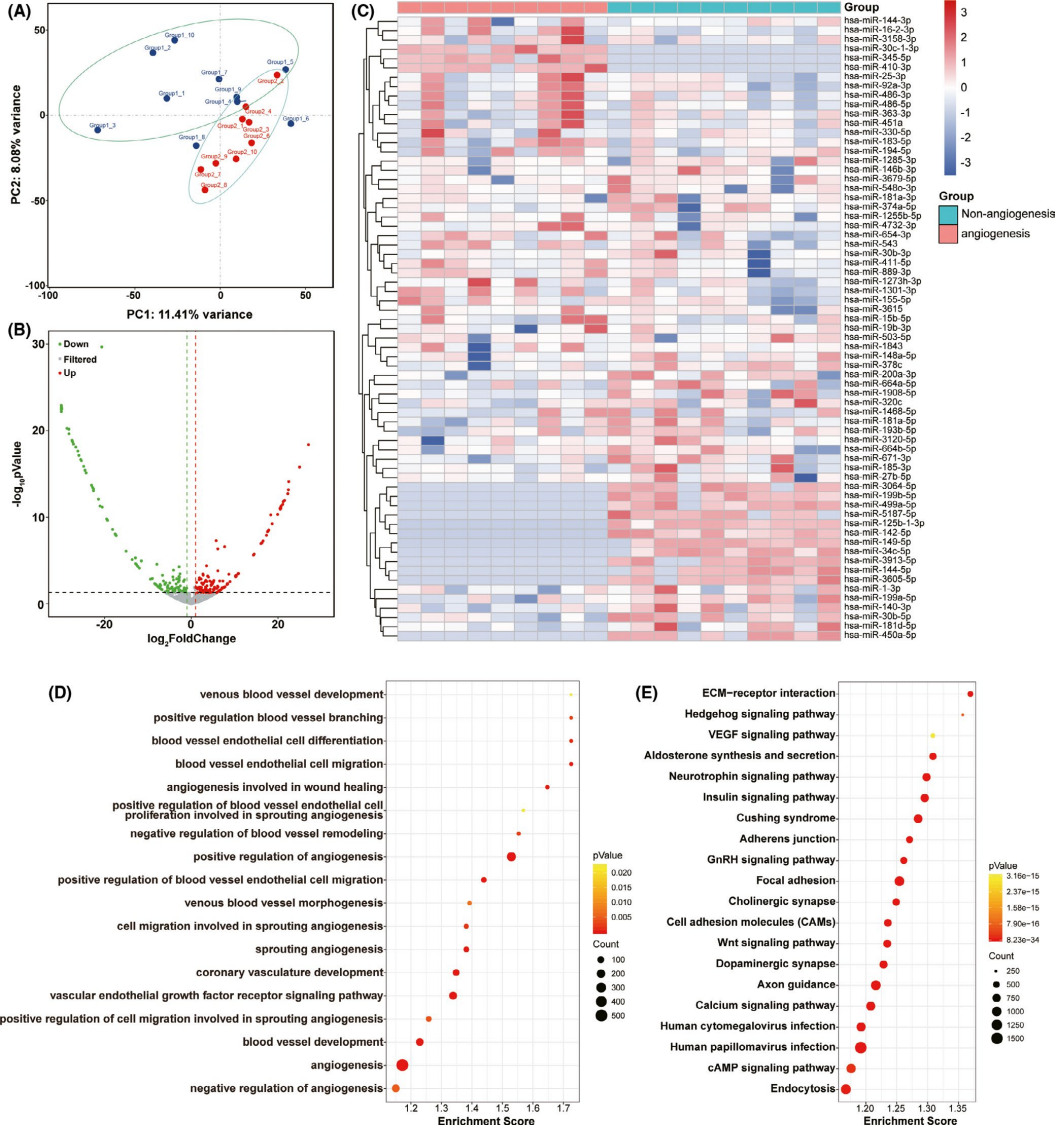
Sequencing data analysis within Angiogenesis and Non‐Angiogenesis Groups. (A) Principal component analysis (PCA) of the two groups. The PCA plot shows PC1 and PC2 indicating 11.4% and 8.08% of the total variance, respectively. (B) Volcano plot showing miRNAs differentially expressed between two groups. Normalized fold change and P values were used to construct the volcano plots. The horizontal and vertical lines represent P value and fold change, respectively. The red and green dots represent statistically significantly upregulated and downregulated miRNAs. The gray dots represent no statistically significantly altered miRNAs. (C) Heat map of miRNA sequencing expression data from CSF samples of individuals in angiogenesis group (*n =* 9) and non‐angiogenesis group (*n =* 10). Red indicates upregulation, and blue indicates downregulation. (D) GO analysis of sequencing data. The bubble pattern showing the biological processes related to angiogenesis which *p <* 0.05. (E) KEGG analysis of sequencing data. The bubble pattern showing the top 20 enrichment pathways with Enrichment score, gene count, and *p* value

We used principal component analysis (PCA) to examine the differences between the miRNAs contained in each array data set. Simplifying this highly multidimensional data set into two‐dimensional components enables unbiased comparison and visualization of total miRNA between samples. The results showed that the angiogenesis group and non‐angiogenesis group could be distinguished significantly at the miRNA expression level. This result indicated that angiogenesis in MMD patients may be closely related to miRNA expression in CSF, and the miRNA content in CSF may represent a potential biomarker for angiogenesis prediction (Figure 2A).

### 3.3 Differential miRNA analyses between two groups

To screen candidate miRNAs in CSF, we analyzed the differences in miRNA in the CSF between the angiogenesis group (*n* = 9) and the non‐angiogenesis group (*n* = 10) through small RNA sequencing. In CSF, we detected 888 known miRNAs and 1,070 novel miRNAs. A total of 138 miRNAs were significantly different between the two groups (Figure 2B), specifically, 75 upregulated miRNAs and 63 downregulated miRNAs with the threshold of *p* values <0.05 and |fold change| >2.0. Thirty‐seven upregulated and 30 downregulated known miRNAs in the angiogenesis group that were detected in more than 80% the samples in at least one group were displayed with a heat map to show the expression status of miRNAs in each sample between the two groups (Figure 2C).

### 3.4 Bioinformatics analysis for all differential miRNAs

To further elucidate the potential biological functions of all differential miRNAs, target genes of differential miRNAs were predicted, and functional enrichment analysis was performed for the target genes. We performed GO analysis and KEGG analysis on target genes of all differential miRNAs. According to the GO analysis results, 2,653 biological process (BP) terms, 427 cellular component (CC) terms, and 852 molecular function (MF) terms were significantly enriched. The results showed that many biological processes related to angiogenesis were significantly enriched, including angiogenesis, positive regulation of angiogenesis, blood vessel endothelial cell differentiation, and venous blood vessel development (Figure 2D). According to the KEGG enrichment analysis, the top 20 pathways are shown in Figure 2E. The enrichment pathway results showed that the VEGF signaling pathway, Wnt signaling pathway, and cAMP signaling pathway, which can regulate angiogenesis and cell adhesion, were significantly enriched. Functional enrichment analysis of target genes further demonstrated that angiogenesis in MMD patients may be closely related to differential miRNAs in CSF. The small RNA‐sequencing data are available in Gene Expression Omnibus with the accession number: GSE168469.

### 3.5 Candidate miRNAs screening

**FIGURE 3:**
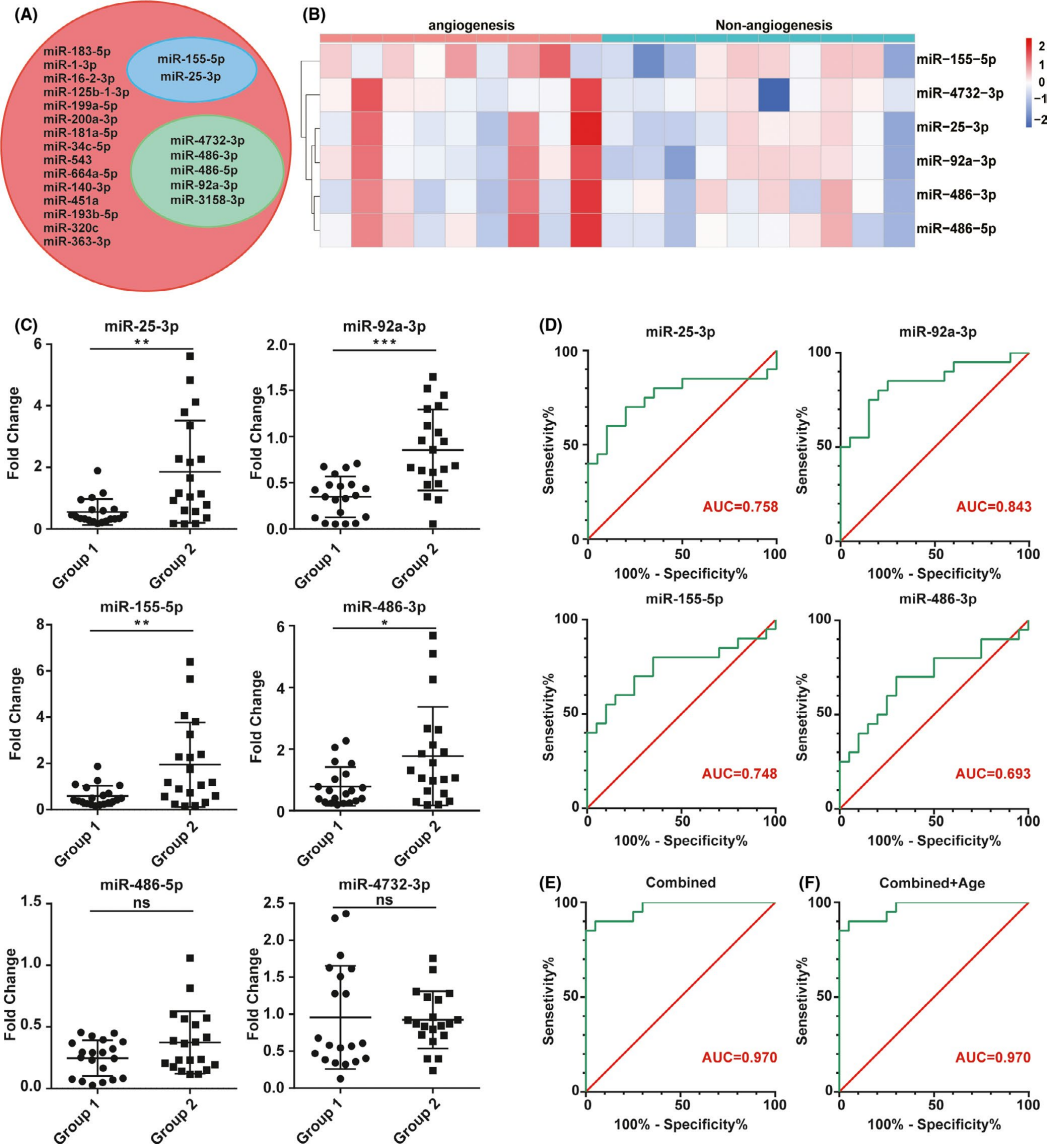
Validation of candidate miRNAs by quantitative reverse‐transcription polymerase chain reaction (qRT‐PCR) and ROC analysis of significant miRNAs. (A) Candidate miRNAs screening by functional analysis (Green) and literature review(Blue). (B) Heat map of miRNAs that differ significantly between the two groups. (C) Expression levels of the candidate miRNAs in CSF among angiogenesis group (*n =* 20) and non‐angiogenesis group (*n =* 20). (D) The AUC (Red numbers) of significant miRNAs being analyzed by ROC curves. (E) ROC analysis of combination of the 4 miRNAs. The 4 combined miRNAs had a stronger predictive value than a single miRNA. (F) ROC analysis of combination of the 4 miRNAs and age. The difference in age does not affect this prediction model. Group 1: angiogenesis; Group 2: non‐angiogenesis; AUC, area under the curve; ns, no sense; *: *p <* 0.05; **: *p <* 0.01; ***: *p <* 0.001

After applying the filtering criteria described in the methods, we identified 22 miRNAs (Figure 3A). Five miRNAs related to angiogenesis were identified through functional analysis (Figure 3A, Green), and 2 were obtained through a literature review.^25^, ^26^ (Figure 3A, Blue). The q value of hsa‐miR‐3158‐3p was q=0.12, so it was not included in the subsequent analysis and verification, and the q value of other 6 miRNAs was q≤0.1. Hence, we selected 6 miRNAs (miR‐25‐3p, miR‐486‐5p, miR‐486‐3p, miR‐155‐5p, miR‐4732‐3p, and miR‐92a‐3p) for further verification by quantitative RT‐PCR. The heat map indicates that the expression of miRNAs is different in the angiogenesis group and the non‐angiogenesis group (Figure 3B).

### 3.6 Validation of candidate microRNAs by qRT‐PCR

The expression of the 6 candidate miRNAs from the screening phase was further analyzed in 20 angiogenesis group patients and 20 non‐angiogenesis group patients using qRT‐PCR. As shown in Figure 3C, four miRNAs (miR‐25‐3p, miR‐92a‐3p, miR‐486‐3p, and miR‐155‐5p) out of the 6 were still upregulated in the angiogenesis group (*p* < 0.05). In general, these differential miRNAs may be related to the establishment of collateral circulation after EDMS.

### 3.7 Diagnostic value of the identified miRNA signature in CSF

Receiver operating characteristic curve analysis was performed to evaluate the diagnostic value of the four identified miRNAs for the establishment of collateral circulation. First, we performed ROC analysis on a single miRNA. As shown in Figure 3D, the ROC curve demonstrated that the AUC was 0.758 for miR‐25‐3p, 0.693 for miR‐486‐3p, 0.748 for miR‐115‐5p, and 0.843 for miR‐92a‐3p with 95% confidence index (CI), sensitivity and specificity, as shown in Table S2. Next, we combined the four miRNAs and constructed a 4‐miRNA panel for diagnosing the establishment of collateral circulation. The ROC curve demonstrated that the AUC was 0.97 (95% CI: 0.926–1.00; sensitivity = 85%, specificity = 100%) (Figure 3E). The combination of four miRNAs was relatively higher than each of the four miRNAs, which further indicated better diagnostic ability of the panel compared with single miRNAs. Due to the significant difference in age between the two groups, we combined four miRNAs and age for ROC analysis (Figure 3F). The ROC analysis demonstrated that the shape of the curve and AUC were exactly the same as the ROC curve, which combined four miRNAs. The results indicated that the difference in age does not affect this prediction model.

### 3.8 Bioinformatics analysis of selected miRNAs and their targets

**FIGURE 4:**
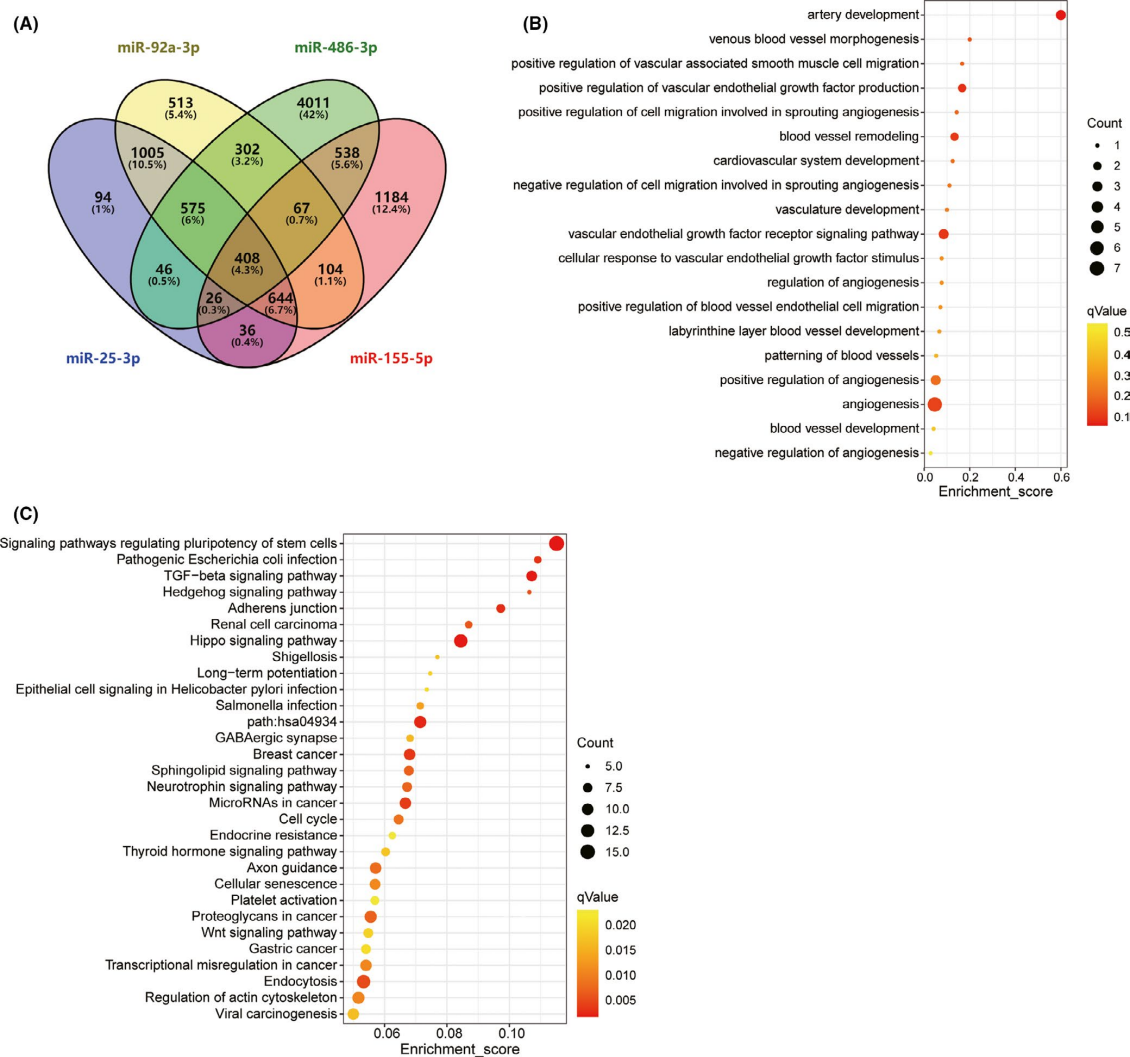
Bioinformation analysis of sequencing data and significant miRNAs. (A) Venn diagram analyses of four independent databases (TargetScan, TargetMiner, miRDB, and miRTarBase) reveal possible downstream targets of the 4 significant miRNAs. Venn diagram shows 408 intersection genes of the four miRNAs. (B) GO analysis of intersection genes. The bubble pattern showing the biological processes related to angiogenesis which *p <* 0.05. (C) KEGG analysis of intersection genes. The bubble pattern showing the top 30 enrichment pathways with Enrichment score, gene count, and *p* value

To further understand the potential biological functions of the four miRNAs, we identified the functions of target genes using GO and KEGG pathway enrichment analyses. According to the target gene prediction, we obtained 2,834 downstream target genes for hsa‐miR‐25‐3p, 5,973 genes for hsa‐miR‐486‐3p, 3,007 genes for hsa‐miR‐155‐5p, and 3,618 genes for hsa‐miR‐92a‐3p. VENN analysis demonstrated that 408 mRNAs can be used as downstream target genes of the above four miRNAs (Figure 4A and Table S3). Next, 408 downstream target genes were used for GO analysis and KEGG pathway enrichment analyses. According to the GO analysis results, 1,202 BP terms, 302 CC terms, and 371 MF terms were significantly enriched in downstream target genes. We also selected the biological processes related to angiogenesis, and the results indicated that many biological processes related to angiogenesis were significantly enriched, including artery development, blood vessel remodeling, and angiogenesis (Figure 4B). The top 30 pathways obtained with the KEGG analysis are shown in Figure 4C. The results indicated that the most significant dysfunctional pathways were in signaling pathways regulating the pluripotency of stem cells. There are also some signaling pathways, such as the Hippo signaling pathway, the adherens junction, and the TGF‐beta signaling pathway, involved in the biological processes of angiogenesis and cell adhesion.

## 4 DISCUSSION

In this study, we analyzed whether patients with moyamoya disease developed angiogenesis after bypass operation. First, we divided the two groups of patients into an angiogenesis group and a non‐angiogenesis group according to DSA follow‐up results at 6 months after surgery. Next, we performed miRNA sequencing on the CSF of the two groups of patients and filtered 4 differential miRNAs through qRT‐PCR. Finally, we performed GO and KEGG analyses on the differential miRNAs. We identified a total of 4 miRNAs as a signature associated with angiogenesis in MMD patients who received combined STA‐MCA bypass and EDMS surgery. Furthermore, we were the first to validate that miR‐92a‐3p, miR‐486‐3p, miR‐25‐3p, and miR‐155‐5p in CSF may serve as novel potential biomarkers for the diagnosis of angiogenesis after EDMS. In addition, we indicated that miR‐92a‐3p, miR‐486‐3p, miR‐25‐3p, and miR‐155‐5p were associated with several signaling pathways, such as the VEGF and Hippo signaling pathways, promoting angiogenesis in MMD patients with bypass operation.

MicroRNAs, as factors involved in the biological process of angiogenesis, have been widely reported. Zeng et al. demonstrated that miR‐25‐3p promotes angiogenesis in colorectal cancer.^27^ Liu et al. demonstrated that miR‐92a‐3p regulates angiogenesis in recipient ECs through a THBS1‐dependent mechanism.^28^ Zhou et al indicated that miR‐155‐5p could control melanoma angiogenesis.^26^

This study indicated that the 4 miRNAs can predict the angiogenesis of patients who suffered EDMS in the combination of logistic regression. Although a single miRNA is meaningful for the prediction of angiogenesis, the biological process of angiogenesis must be multifaceted; therefore, we combined four miRNAs for analysis. The results demonstrate that combining four miRNAs is a better prediction than combining a single miRNA. Additionally, we found that there was a difference in age between the two groups; so, we added the factor of age to the four miRNAs for multivariate analysis. It is very interesting to found that the result after adding age is the same as when not adding; therefore, we believe that age has no effect on angiogenesis in this predictive model.

The process of angiogenesis includes the following four aspects: 1) proteolysis of the basement membrane, 2) migration and chemotaxis at the capillary tip, 3) cell proliferation, and 4) increased permeability through gaps.^29^ Several articles have reported that miRNAs can promote cell proliferation and migration. Ouyang et al. demonstrated that miR‐25‐3p can promote the proliferation of HepG2.2.15 cells.^30^ Zhu et al. indicated that miR‐671‐5p could facilitate PCa cell proliferation and migration *in vitro* and *in* *vivo*.^31^ Li et al. found that miR‐301a promotes cell proliferation and migration in lung tumorigenesis by suppressing Runx3.^32^

According to the GO analysis of the above four miRNAs, we found that these 4 miRNAs are involved in many biological processes related to angiogenesis, such as artery development, positive regulation of cell migration involved in sprouting angiogenesis and blood vessel remodeling. Some signaling pathways of KEGG analysis have been widely reported. Pulkkinen et al. demonstrated that BMP6 modulated VEGF signaling by regulating VEGFR2 expression and acted through the Hippo signaling effector TAZ, which is known to regulate cell survival/proliferation.^33^ Chavali et al. demonstrated that hypoxia induced increases in oligodendrocyte precursor cell numbers, vessel density, and endothelial cell expression of the Wnt signaling pathway targets Apcdd1 and Axin2 in white matter.^34^ The importance of the TGF‐β signaling pathway in angiogenesis has been reported in recent decades.^35^, ^36^, ^37^ Hence, recent evidence has suggested that these 4 miRNAs are highly related to angiogenesis.

Why did we choose miRNAs from CSF instead of serum as biomarkers for our study? First, angiogenesis between the temporal muscle and cerebral cortex after EDMS occurs in the CSF environment. It is possible that key factors in the CSF play an important role in the biological communication between the vascularized grafts and the cerebral cortex to initiate and promote the angiogenesis process. MMD is a central nervous system (CNS) disease, and CSF is the biological fluid in closest contact with the CNS. Using factors in CSF as biomarkers can minimize sources of preanalytical variability. Therefore, CSF represents the gold standard fluid for biomarker identification for neurological diseases.^38^, ^39^, ^40^

According to the published literature, half of the adult patients did not form good collaterals, which means the indirect bypass surgery was ineffective for these patients but increased the procedure‐related complications that we mentioned before. This study was able to provide a tool to predict the possibility of angiogenesis in MMD patients after EDMS surgery. So for these patients, we could produce a rapid assay kit for detecting the targeted miRNAs in CSF obtained from MMD patients by lumbar puncture before surgery. Therefore, we tested the CSF with the kit and considered whether to perform EDMS surgery on the patients based on the results. This method can effectively reduce the surgical complications by choosing a more reliable bypass procedure.

Although these results are encouraging, some limitations of our study should be realized. First, the number of patients enrolled in our study was relatively small. Second, we did not explore the mechanism by which miRNAs promote angiogenesis. In the future, we will further explore the effect of miR‐92a‐3p, miR‐486‐3p, miR‐25‐3p, and miR‐155‐5p on the biological process of angiogenesis by such experiments as the CCK8 assay, tube formation assay, and migration assay. In addition, we will attempt to follow‐up with these patients for longer periods.

## 5 CONCLUSIONS

In summary, we demonstrated that miR‐92a‐3p, miR‐486‐3p, miR‐25‐3p, and miR‐155‐5p in CSF could serve as novel potential biomarkers for the prediction of angiogenesis after EDMS in adult MMD patients.

## CONFLICT OF INTEREST

The authors declared no potential conflicts of interest with respect to the research, authorship, and/or publication of this article.

## AUTHOR CONTRIBUTIONS

Gang Wang and Yunyu Wen conducted most of the benchwork, assembled the results, and wrote the manuscript. Siyuan Chen, Guozhong Zhang, Mingzhou Li, Shichao Zhang, and Songtao Qi performed clinical specimens. Yunyu Wen performed total RNA extraction and qRT‐PCR. Gang Wang and Wenfeng Feng provide funds and ideas. All authors contributed to the article and approved the submitted version.

## Supporting information

Tab S1

Tab S2

Tab S3

## Data Availability

The data that support the findings of this study are available from the corresponding author upon reasonable request.
